# A three-dimensional visualization of the full-field surgical region based on thin-slice MRI: A helpful approach for simultaneously guiding tumor resection and perforator flap elevation

**DOI:** 10.3389/fsurg.2022.984892

**Published:** 2022-09-22

**Authors:** Lei Cui, Wei Q. Jiang, De K. Zhang, Gao F. Wang, Yu D. Han, Wen W. Pu, Yan Shao, Lin L. Guo, Ran Tao, Yan Han

**Affiliations:** ^1^ Department of Plastic and Reconstructive Surgery, 1st Medical Center of Chinese PLA General Hospital, Beijing, China; ^2^ Department of Radiology, 1st Medical Center of Chinese PLA General Hospital, Beijing, China; ^3^ Department of Pathology, 1st Medical Center of Chinese PLA General Hospital, Beijing, China; ^4^ Plastic Surgery Hospital (Institute), CAMS, PUMC, Beijing, China

**Keywords:** thin-slice MRI, three-dimensional reconstruction, *en bloc* tumor resection, perforator flap elevation, soft tissue sarcoma (STS), skin squamous cell carcinoma (SCC)

## Abstract

**Background:**

The goal of the current study was to explore the application of preoperative three-dimensional reconstruction (3DR) based on thin-slice magnetic resonance imaging (MRI) in the simultaneous guidance of *en bloc* tumor resection and adjacent perforator flap elevation.

**Methods:**

The prospective cohort included 35 patients diagnosed with either soft tissue sarcoma or squamous cell skin cancer between 2019 and 2021. The preoperative 3DR based on thin-slice MRI illustrated the spatial anatomical relationship among the tumor, underlying muscle, adjacent perforator vessels, and bone around the surgical region. The accuracy of preoperative imaging data was verified by intraoperative vessel dissection and postoperative pathological measurements.

**Results:**

Tumor size from 3DR data showed relatively high concordance rates with pathological measurements within the 95% limits of agreement. An average of three perforators (range: 1–7) with a mean diameter of 0.32 cm (range: 0.18–0.74 cm) from the 3DR were present in our study. The average distance between tumor boundary and perforator piercing sites on the 3DR was 2.2 cm (range: 1.2–7.7 cm). The average length of artery perforator coursing along the subcutaneous tissue was 5.8 cm (range: 3.3–25.1 cm). The mean flap harvest time was 55 mins (range: 36–97 min). The average flap size was 92.2 cm^2^ (range: 32–126 cm^2^). One perforator flap occurred distal partial necrosis.

**Conclusion:**

A thorough understanding of anatomical structures in the surgical region according to full-field 3DR based on thin-slice MRI can improve the performance of radical resection of the tumor and adjacent perforator flap transfer, especially for junior surgeons with a poor experience.

## Introduction

Compared with other types of malignant tumors, both soft tissue sarcomas (STSs) and skin squamous cell carcinomas (SCCs) have a higher risk of local recurrence, lower risk of distant metastasis, and insensitivity to chemotherapy and radiotherapy. Therefore, complete excision of the tumor with negative microscopic margins is a primary prognostic factor for local recurrence (1–3). It is common for plastic surgeons to perform radical tumor resection with wider surgical margins and wound defect coverage during the same operation. A perforator-pedicled propeller flap (PPPF) is a versatile technique with many benefits, such as adequate blood supply, like-with-like repair, minimal donor site morbidity, and no extra intraoperative patient’s position alteration (4–6). Given the abundance of musculocutaneous perforators for the overlying skin in trunks and extremities, PPPFs serve as a primary workhorse for treating the defects caused by wide resection of tumors in these regions (7–10).

Preoperative imaging assessment plays an important role in determining the extent of tumor resection and the distribution of the surrounding perforator vessels. Magnetic resonance imaging (MRI) is more sensitive than computed tomography and ultrasound for imaging soft tissue lesions. Kwon et al. (11) demonstrated that there was a relatively high concordance in tumor thickness between MRI and histopathology in patients with oral tongue squamous cell carcinoma. With the progressive development of intraoperative MRI, surgeons have tried to use intraoperative MRI to guide the resection of soft tissue sarcoma to reduce the re-resection rates (12, 13). On the other hand, MRI with or without enhanced contrast has made noteworthy progress in perforator mapping in the past decades. Some studies have demonstrated that magnetic resonance angiography (MRA) can serve as an alternative to computed tomography angiography (CTA) (14–16). Color duplex sonography (CDS), a noninvasive perforator imaging tool with a lower cost, can provide anatomical and hemodynamic information about small vessels and be reproducible during surgery as well (17–19). Otherwise, the diagnostic performance of CDS is unstable because it requires skilled operators with a great deal of knowledge about vascular anatomy. In summary, MRI has similar accuracy in perforator evaluation to CTA or CDS, especially in those with a diameter greater than 1 mm qualifying for nourishing the PPPF. Hence, in the present study, we chose thin-slice MRI to simultaneously obtain anatomic information referring to tumor infiltration and adjacent perforator vessel distribution.

Even though MRI can clearly display imaging related to tumors and perforating vessels, surgeons have no stereoscopic perspectives on the anatomic relationship among the tumor, underlying muscle, and adjacent perforator vessel, which calls into question whether the radical resection of the tumor would damage the perforators for supplying the designed flap. The approach of three-dimensional (3D) digitalized visualization is supposed to resolve these problems. Until now, there has been many research studies on 3D reconstruction. However, previous studies mainly focused on a single objective, for example, 3D reconstruction based on CTA or MRA for thin vessels in various surgical fields (20) and 3D reconstruction of the extent of tumor invasion using CT, MRI, or data fusion (21, 22). Also, the use of 3D virtual planning to guide flap harvesting has been previously reported, especially for osteomyocutaneous microvascular fibular free flaps repairing head and neck defects after mandibular tumor resections, which is different from tumor resection and reconstructed by local tissue (23, 24). In our study, we simultaneously paid attention to wide resection of the tumor and adjacent PPPF harvesting. We aimed to demonstrate the comprehensive information of full-field surgical region by three-dimensional reconstruction (3DR) based on thin-slice MRI data, which involved tumor size, the depth of tumor infiltration, the origin and course of perforator vessel, and the relationship between tumor and perforator. The 3D visualization is supposed to assist clinicians in perceiving the surgical area from multiple orientations, increasing the accuracy of radical resection of the tumor, and facilitating the design of PPPF.

## Patients and methods

**Figure 1:**
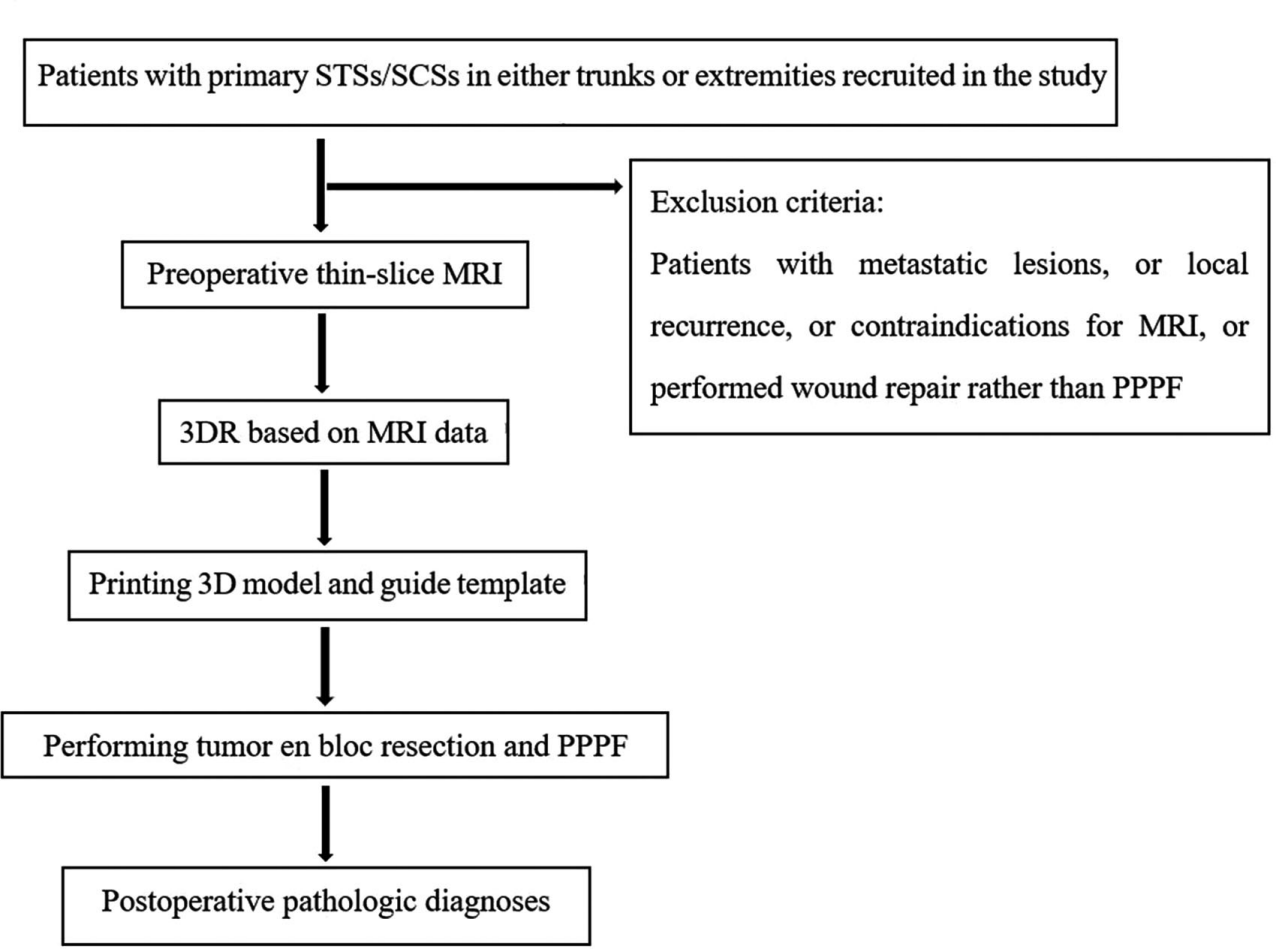
Workflow of our present study.

The study was approved by the Medical Ethics Committee of the Chinese PLA General Hospital. From October 2019 to January 2021, 35 patients diagnosed with primary STSs or SCCs in either trunk or extremities were included in the current study. Exclusion criteria were patients with metastatic lesions, local recurrence, contraindications for MRI, or wound repair rather than PPPF. The multidisciplinary team comprised two senior MRI radiologists with more than 10 years of experience in malignant soft tissue tumors and vascular diagnosis, a bioengineer optimizing the digitalized reconstruction, and a surgical group of plastic surgeons under the supervision of the corresponding author. The workflow is illustrated in Figure 1.

### MRI protocol

All patients, in positions consistent with surgical procedures, underwent MRI on a 3.0 T clinical scanner (GE Healthcare, Beijing, China) using a body or cardiac coil. To explore the perforating vessels, the MRI scan region was extended from 10 cm above the upper tumor margin to 10 cm below the lower border of the tumor. Scanning began with the acquisition of the three-plane localizer. The conventional sequences included axial and coronal single-shot fast spin echo (SSFSE) sequence, T1-weighted sequence, T2-weighted sequence, and diffused-weighted imaging (DWI) sequence. Enhancing imaging was acquired by injecting peripherally gadopentetate dimeglumine (0.1 mmol per kg of bodyweight; Consun, China) at a rate of 1 ml/s followed by a saline flush with the same injection rate. In our practice, the liver acquisition with accelerated volume acquisition (LAVA) sequence was used for 3DR because of the high spatial resolution and continuous thin slices. The parameters are as follows: 3.9 ms repetition time (TR)/1.67 ms echo time (TE)/12°–15° flip angle, 125 kHz bandwidth, 2 mm section thickness reconstructed at 1 mm intervals using two-fold zero interpolation (ZIP 2), 512–128 × 256 matrix, and a parallel acceleration factor of 2 (Table 1).

**Table 1:** MRI parameters for three-dimensional reconstruction in the current research.

Scanner	GE signal
Patient position	Surgical position
Contrast	Gadopentetate dimeglumine
Key sequences	LAVA-sequence
Imaging plane	Delay phase
TR/TE	3.9/1.67
Flip angle	12°–15°
Slice thickness	2.0 mm reconstruction at 1.0 mm intervals
Matrix	512–128 × 256
Bandwidth	125
Field of view	40 cm
Coverage	10 mm above and below from relative tumor margin

LAVA, liver acquisition with accelerated volume acquisition; TR/TE, repetition time/echo time.

### Three-dimensional reconstruction and printed model

**Figure 2:**
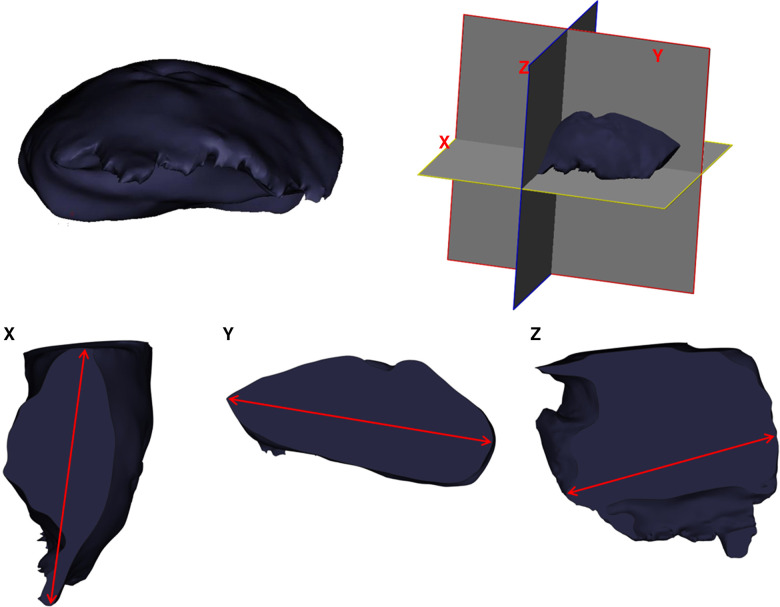
Diagram demonstrating the measurement of tumor size on 3DR models and surgical resected specimen.

The reconstruction process began by converting Digital Imaging and Communications in Medicine (DICOM) data to Mimics Medical v21 software for medical image segmentation (Materialise Inc., Belgium). First, we established the respective segmentation masks for tumors, vessels, muscles, and bones. Subsequently, the masks were converted into 3D meshes. Further manipulation included measuring the caliber of perforating vessels, the distance between the tumor boundary and piercing location of perforating vessels, and the length of the vascular pedicle. The protocol for tumor size measurement on the 3DR models is as follows: the X, Y, and Z reference lines were defined as the longest diameters in longitudinal, latitudinal, and vertical orientations, respectively, in accordance with the routine pathological methods and rules (Figure 2). The mesh was then exported to the .stl ﬁle format, which was further processed to print a 3D surgical guide template marking the projection points of the vascular pedicles, a 2 cm distance from the tumor boundary (Figure 4).

**Figure 3:**
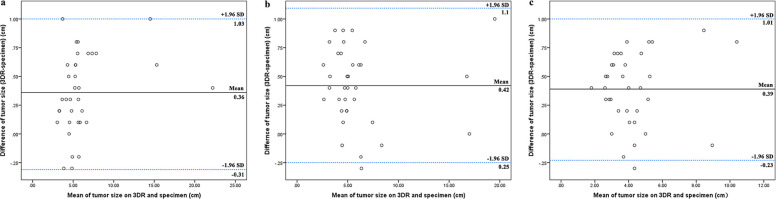
Correlation of tumor size between 3DR data and the specimens. The longest tumor diameters measured on 3DR in the horizontal orientation (**A**), the vertical orientation (**B**), and the saggital orientation (**C**) were compared to the corresponding distances in the pathological specimen. The Bland–Altman plot on each comparison demonstrated relatively high concordance rates within the 95% limits of agreement.

**Figure 4:**
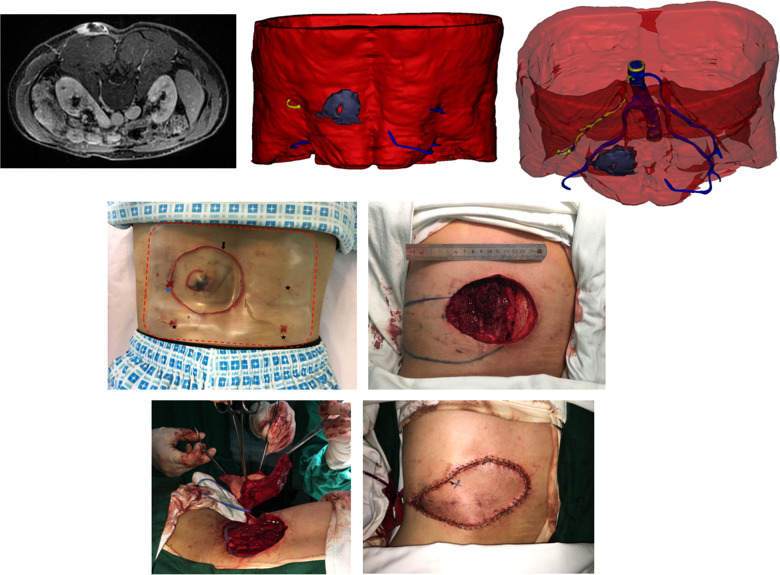
(Above, left) preoperative MRI showing the tumor infiltrating the underlying muscle and adjacent perforator. (Above, middle) 3DR showing the surgical anatomy involving the tumor, underlying muscle, and four perforators. The PPPF was planned based on the yellow perforator. (Above, right) Transparent image showing perforators and main source arteries. (Center, left) Skin incision (black arrow) at 2 cm distance from the tumor boundary and the projection of four perforators according to the surgical guide templates (red dotted line) preoperatively. The blue star represents the perferred yellow perforator, and the three black stars represent the remaining blue perforators. (Center, right) Wound after *en bloc* tumor resection and flap design. The circle represents the piercing site of the superiorly left perforator. The star represents the piercing site of the inferiorly left perforator. (Below, left) harvesting the flap. (Below, right) rotation of the flap for 130° to cover the wound and direct closure of the donor site.

### Surgery

Prior to the surgeries, the skin incisions and projection of the preferred perforators were outlined according to the surgical guide templates. The piercing sites of preferred perforators were also confirmed using color duplex sonography preoperatively. First, the surgeons performed *en bloc* tumor resection. Sequentially, the wound defects were reconstructed by adjacent PPPFs nourished by the marked perforating vessel. The selection criteria for the optimal perforator are specifically set forth in the “Discussion” section. The surgical findings serve as the “gold standard” for identifying the perforator location. During the surgery, we dissected the exact site of perforator piercing from the deep fascia, which was used as the rotation point of the propellor. It was considered positive when the actual piercing site of the preferred perforator was detected within 10 mm distance from the corresponding spot with a symbol marked on the skin based on the surgical guide template, ;otherwise, it was considered negative (Figure 4). The donor sites were repaired by either direct closure or split-thickness skin grafting.

### Pathological assessment

Pathological diagnosis is considered as the “gold standard” for tumor size measurement. The routine X, Y, and Z reference lines reported by pathologists were in the same manner as that described in the previous “Three-dimensional reconstruction and printed model” section (Figure 2).

### Data analysis

Descriptive statistics were calculated to summarize patient characteristics and PPPF information. The tumor size in pathological diagnosis was compared to that measured on the 3DR model using the Bland–Altman plot. Sensitivity was calculated to compare the perforator courses on 3DR models to surgical findings. All statistical analyses were performed using IBM SPSS software version 23.0 (IBM Corp., Armonk, NY).

## Results

### Patient demographics

A total of 35 patients (24 men, 11 women) with a median age of 42 years (range: 27–68) were enrolled in our research. These 35 pathologically confirmed malignant soft tissue tumors included 17 dermatofibrosarcoma protuberans (DFSP), 8 SCCs, 7 liposarcomas, and 3 rhabdomyosarcomas. Histologic grades 1, 2, and 3 accounted for 34.3%, 48.6%, and 17.1%, respectively. Of 35 tumors, 14 (40.0%) were located in the posterior trunk, 9 (25.7%) were located in the anterior trunks, 8 (22.9%) were located in the lower extremities, and 4 (11.4%) were located in the upper extremities. All patients underwent *en bloc* tumor resection and PPPF to cover the wound defect, followed by either primary closure (91.4%) or skin grafting to the donor site (8.6%). The patient demographics are summarized in Table 2.

**Table 2:** Patient demographics.

characteristic	No. (%)
No. of patients	35
Age, years	
Mean (range)	42 (27–68)
Gender	
Male	24 (68.6%)
Female	11 (31.4%)
Histologic type	
DFSP	17 (31.4%)
Skin SCC	8 (22.9%)
Liposarcoma	7 (20.0%)
Rhabdomyosarcoma	3 (8.57%)
Histologic grade (G)	
G1	12 (34.3%)
G2	17 (48.6%)
G3	6 (17.1%)
Tumor location	
Anterior trunk	9 (25.7%)
Posterior trunk	14 (40.0%)
Lower extremity	8 (22.9%)
Upper extremity	4 (11.4%)
*En bloc* tumor resection	35 (100.0%)
PPPF	35 (100.0%)
Donor site closure	
Direct closure	32 (91.4%)
Split-thickness skin grafting	3 (8.6%)

DFSP, dermatofibrosarcoma protuberans; SCC, squamous cell carcinoma; PPPF, perforator-pedicled propeller flap.

### Concordance assessment

Tumor sizes measured on 3DR models on X, Y, and Z reference lines were compared to the relative territory in the pathological specimen. The Bland–Altman plot for each comparison demonstrated relatively high concordance rates within the 95% limits of agreement (Figure 3).

Table 3 shows the main source arteries of the 35 PPPFs displayed on the 3DR models. An average of three perforators (range: 1–7) with a mean diameter of 0.32 cm (range: 0.18–0.74 cm) were present in our current study. The average distance between the tumor boundary and the exact location of perforator piercing from the deep fascia was 2.2 cm (range: 1.2–7.7 cm). The average length of the artery perforator coursing along the subcutaneous tissue was 5.8 cm (range: 3.3–25.1 cm). These above values were obtained from the 3DR without intraoperative identification.

**Table 3:** PPPF characteristics.

Detail	Value
Source artery	
Lumbar artery	26
Superior gluteal artery	10
Posterior intercostal artery	12
Anterior intercostal artery	3
Thoracodorsal artery	5
Thoracoacromial artery	4
Lateral thoracic artery	2
Superficial cervical artery	2
Dorsal scapular artery	6
Lateral sacral artery	1
Internal thoracic artery	2
Musculophrenic artery	3
Inferior epigastric artery	10
Superior epigastric artery	4
Superficial epigastric artery	2
Descending branch of LCFA	2
Ascending branch of LCFA	1
MCFA	1
Profunda femoral artery	2
Superficial femoral artery	4
Peroneal artery	2
Posterior tibial artery	1
Anterior tibial artery	1
Profunda brachii artery	3
Radical collateral artery	2
Brachial artery	3
Superior ulnar collateral artery	1
Radical artery	1
Ulnar artery	1
Number of the perforators per patient	
Mean (range)	3 (1, 7)
Caliber of the perforators (cm)	
Mean (range)	0.32 (0.18, 0.74)
Distance between tumor boundary and piercing location, cm	
Mean (range)	2.2 (1.2, 7.7)
Length of vessel pedicle (cm)	
Mean (range)	5.8 (3.3, 25.1)
Flap size (cm^2^)	
Mean (range)	92.2 (32–126)
Flap harvest time (min)	
Mean (range)	55 (36, 97)

LCFA, lateral circumflex femoral artery; MCFA, medial circumflex femoral artery.

The average flap size was 92.2 cm^2^ (range: 32–126 cm^2^). The mean flap harvest time was 55 mins (range: 36–97 min). During the PPPF elevations, all actual piercing sites of preferred perforators were visualized and verified at a distance of within 10 mm from the marked sites without false-positive findings; hence, we concluded that there was a 100% positive predictive value in terms of perforator vessel evaluation by 3DR based on the thin-slice MRI sequence.

Two patients presented with postoperative complications (5.7%). One patient appeared with venous congestion. The flap healed uneventfully after local subdermal heparinization. The distal partial necrosis occurred in the other flap due to mixed vascular issues. After conservative treatment, the patient underwent a secondary operation with debridement and a split-thickness skin graft (2.9%).

## Case reports

### Case 1

A 47-year-old man was diagnosed with DFPS in the left lateral lumbar region (Figure 4, Supplementary Video S1). MRI showed that the tumor had invaded the muscle. 3DR showed a 5.8 × 4.1 × 3.4 cm tumor with muscle infiltration and four perforators adjacent to the tumor originating from the lumbar arteries. The diameters of these perforators are as follows: 0.28, 0.36, 0.41, and 0.44 cm. The distances between tumor boundaries and piercing sites are as follows: 3.5, 6.0, 9.2, and 9.2 cm. The PPPF was planned based on the perforator with a diameter of 0.28 cm and a distance of 3.5 cm. Skin incision at 2 cm distance from the tumor boundary and the projection of four perforators were outlined on the skin according to the surgical guide templates preoperatively. The patient underwent wide excision of the tumor with an 11 × 8 cm wound defect. During surgery, these two left perforators were identified. After ligating the lower perforator, an 18 × 7 cm flap was rotated 130° to resurface the defect. The donor site was closed by primary closure. The flap survived without postoperative complications.

### Case 2

**Figure 5:**
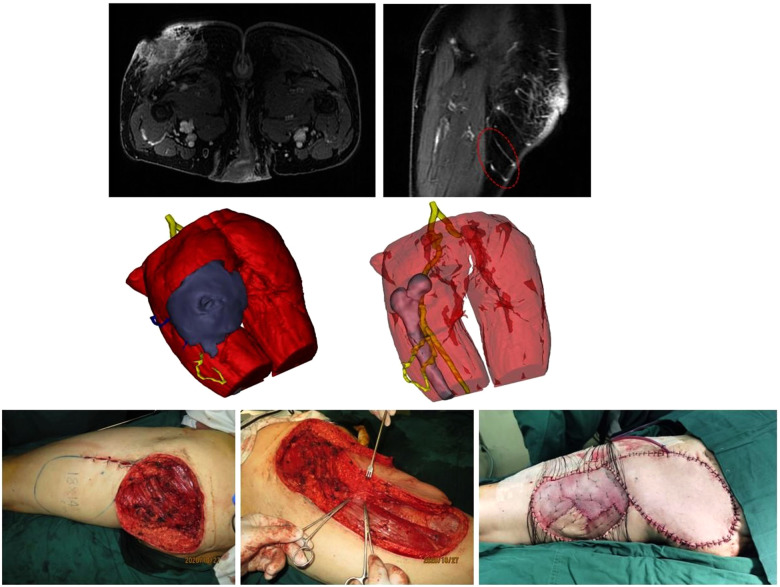
(Above) preoperative MRI showing the tumor infiltrating the underlying muscle (left) and adjcent perforators (right). Red dotted line circling the preferred perforators for PPPF. (Center, left) 3DR showing the surgical anatomy involving the tumor, underlying muscle, and perforators. The PPPF was planned based on the yellow perforators. (Center, right) The yellow perforators were intact after wide excision of the tumor. (Below, left) the wound after *en bloc* tumor resection and the flap design. (Below, median) harvesting the flap. The clamps pointed to the perforators for PPPF. (Below, right) rotation of the flap for 160° to cover the wound and free skin graft to close the donor site.

A 63-year-old man was admitted with sSCC in the left posterior thigh (Figure 5, Supplementary Video S2). MRI showed that the tumor spread extensively along the subdermal tissue and invaded the gluteal muscle. 3DR visualization showed that the tumor with a size of 15.4 × 15.9 × 13.3 cm infiltrated the underlying muscles with an extent of 9.8 × 8.7 × 4.3 cm. There were three main branches distributed around the left femur according to the 3DR. The lower perforators originating from the profunda femoral artery were chosen to supply the PPPF. The patient underwent wide excision of the tumor with a 20 × 24 cm wound defect. A PPPF with a size of 18 × 14 cm was rotated 160° to cover the defect. The donor site was closed by a free skin graft. The flap survived without postoperative complications.

### Case 3

**Figure 6:**
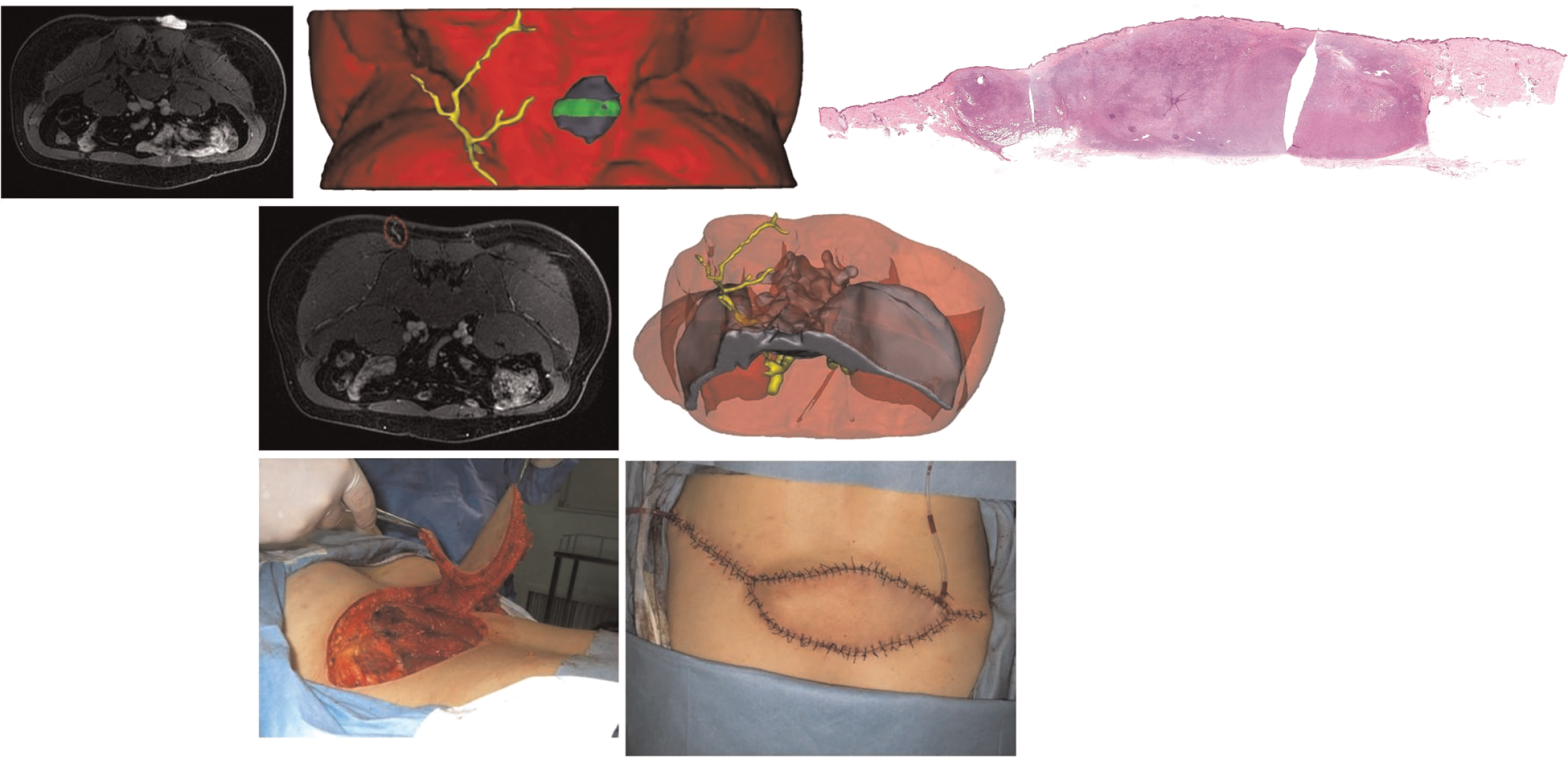
(Above, left) preoperative MRI showing the tumor overlying the thoracolumbar fascia. (Above, median) 3DR showing the surgical anatomy involving the tumor, underlying muscle, and perforators. The green area corresponds to the front MRI picture and later pathological imaging. (Above, right) Integrated H/E (hematoxylin and eosin) image from multiple slices showing the pathological morphology of the whole tumor section. (Center, left) preoperative MRI showing the preferred perforator (red dotted ellipse). 3DR showed that the preferable perforator originated from the lateral sacral artery piercing the second anterior and posterior sacral foramens. (Below, median) Harvesting the flap. (Below, right) rotation of the flap for 160° to cover the wound and direct closure of the donor site.

A 34-year-old man was diagnosed with DFSP in the right lumbosacral region (Figure 6, Supplementary Video S3). The MRI showed that the tumor adhered to the underlying muscle and perforators around the tumor were widespread. 3DR showed a 4.5 × 4.5 × 2.2 cm tumor overlay on the thoracolumbar fascia. The preferable perforator originated from the lateral sacral artery piercing the second anterior and posterior sacral foramina, with a diameter of 0.24 cm and a distance of 5.3 cm from the tumor margin. The patient underwent wide excision of the tumor with a 14 × 8 cm wound defect. A PPPF with a size of 20 × 6.5 cm was rotated 160° to cover the defect. The donor site was closed directly. The flap survived uneventfully.

## Discussion

When it comes to the scanning of soft tissue lesions, MRI is more sensitive than CT for differentiating neoplasms and identifying the extent of cancerous tissue invasion. Moreover, MRI is also more suitable for detecting perforating vessels close to the tumor, which is extremely variable with random tumor sites and originates from inconclusive sources. Therefore, our study only applied MRI to obtain anatomical information regarding cancerous tissue and adjacent perforating arteries. Because there was no additional imaging acquisition, such as CTA for perforators, this approach of thin-slice MRI was time- and cost-saving for the patients. On the basis of thin-slice MRI in our present research, we reconstructed the surgical region consisting of tumors, underlying muscles, adjacent perforators, and bone if necessary.

First, 3DR imaging can display stereoscopic tumors. In addition to the entire tumor, the infiltrated fascia and muscle were also reconstructed and labeled on the surgical template. The surgical guide template marked the incision at a 2 cm distance from the tumor boundary and the projection of deep tissue invasion, which was supposed to enhance the success rate of *en bloc* tumor resection and acquire a better oncologic outcome. Pathological diagnosis is considered the “gold standard” in terms of tumor measurement. Figure 3 shows that tumor size measured by the 3DR models had a significant concordance with the results of the pathological assessment. In case 3, there was a remarkable consistency in the size and morphology of the integrated tumor cross section between hematoxylin and eosin (H/E) staining and MR imaging (Figure 6).

Additionally, it is realizable for surgeons to observe the surgical area from multiple orientations and measure the stereoscopic value according to the 3DR visualization, which could provide more abundant and reliable arguments for selecting targeted perforators. The selection criteria for choosing the preferred perforators in each case are as follows. First, the perforators were reserved after extensive resection of the tumor, which was of paramount importance. In case 2, the above perforator originating from the profunda femoral artery was ruled out because it would be removed after wide local excision (Figure 5, Supplementary Video S2). In other cases, the perforators would be excluded when the main source vessels are located in the surgical area on 3DR visualization. The distance between the tumor margin and piercing site measured by 3DR models was also considered essential for choosing the ideal donor site. In case 1, the right perforators were inappropriate due to the extremely long distance from the tumor margin (Figure 4, Supplementary Video S1). Other rules included a vascular bundle of ≥0.2 cm or an arterial diameter ≥ 0.1 cm and an extensive branching pattern in the subcutaneous tissue. The mean diameter of perforators measured in our current research was 0.32 cm. We analyzed that the delay sequence applied in three-dimensional reconstruction, concurrently representing the arterial and venous images, might result in a value greater than those in other research studies. In our research, the flap with partial necrosis might be caused by the small caliber of the selected perforator (0.18 cm in diameter of the vascular bundle) and the shorter subcutaneous course (3.3 cm). Although these shortcomings were predicted on preoperative 3DR visualization, no other more suitable perforators were available in this case. The intramuscular route was another factor to be considered. The septocutaneous and direct cutaneous perforators were favorable because they would afford the surgeons the relatively easier and straightforward dissection. In our study, 3DR visualization was capable of transparently manifesting the association between perforator courses and the muscles, which appeared to be a prominent advantage over other imaging modalities. Finally, the distinct visualization of main source vessels provided subsidiary evidence for selecting preferred perforators. Although the reconstruction of bone is obscure, their anatomical positions played a role in distinguishing the main vessels. In case 2, three main branches distributed around the left femur are as follows: profunda femoral artery, lateral circumflex femoral artery, and medial circumflex femoral artery. The preferred perforators in this case originated from the profunda femoral artery that was supposed to be intact after wide excision of the tumor (Figure 5, Supplementary Video S2). In case 3, the preferable perforator originated from the lateral sacral artery piercing the second anterior and posterior sacral foramens, which is inconsistent with common knowledge that the inferior trunk defects are treated by the flaps based on the superior gluteal artery perforators (SGAPs) (Figure 6, Supplementary Video S3). 3DR imaging of case 3, optimized for better visualization after the operation, illustrated the major perforators dispersing along the subcutaneous tissue. Since the ipsilateral perforator vessels appeared to have more enlarged diameters due to tumor intervention and most of these vessels were not used in case 3, the data of these unrelated vessels was not added up in the study (Supplementary Video S3).

The depiction of perforating vessels described in Table 3 was derived from preoperative 3DR data. Since surgeons should obey the principle of shielding from cancerous tissues, the distance between the tumor boundary and piercing location of perforating vessels cannot be counted intraoperatively. Otherwise, because the authors advocate that there is no need to skeletonize perforating vessel if the skin island has no issue with pedicle tension after resurfacing the defect, the caliber of perforators could not be ascertained during the operations. Nevertheless, a total of 35 PPPFs were designed based on preoperative 3D visualization and eventually healed without the problem of ischemia. Therefore, we concluded that the sensitivity was 100% for thin-slice MRI detecting perforators greater than 1 mm surrounding the tumors.

The main limitation of the present study was the unsatisfied tissue resolution. The quality of the LAVA-delay sequence used for 3DR was affected by some factors, such as the scanning location, respiratory influence, operating time, and coil type. Although the information acquired from thin-slice MRI was comprehensive, the artifact and blurring contour of muscle prolonged the reconstructive process, especially in the early stage of clinical practice. The parameters of the LAVA-delay sequence should be improved in the following phase.

## Conclusion

We choose the thin-slice MRI as a preoperative imaging approach to simultaneously evaluate the tumor infiltration and adjacent perforator vessel distribution. It is a versatile and elegant strategy to execute full-field digitalized reconstruction regarding surgical areas based on thin-slice MRI. A thorough understanding of anatomical structures in the surgical region prior to the surgery can improve the performance of radical resection of the tumor and adjacent perforator flap elevation, especially for junior surgeons with a poor experience.

## Data Availability

The raw data supporting the conclusions of this article will be made available by the authors without undue reservation.
